# Analysis of AI-Based Single-View 3D Reconstruction Methods for an Industrial Application

**DOI:** 10.3390/s22176425

**Published:** 2022-08-25

**Authors:** Julia Hartung, Patricia M. Dold, Andreas Jahn, Michael Heizmann

**Affiliations:** 1 TRUMPF Laser GmbH, Aichhalder Str. 39, 78713 Schramberg, Germany; 2 Institute of Industrial Information Technology, Karlsruhe Institute of Technology, Hertzstraße 16, 76187 Karlsruhe, Germany

**Keywords:** three-dimensional reconstruction, single view, stacked autoencoder (SAE), generative adversarial network (GAN), U-Net, stacked dilated U-Net (SDU-Net), artificial intelligence, deep learning, hairpin, production

## Abstract

Machine learning (ML) is a key technology in smart manufacturing as it provides insights into complex processes without requiring deep domain expertise. This work deals with deep learning algorithms to determine a 3D reconstruction from a single 2D grayscale image. The potential of 3D reconstruction can be used for quality control because the height values contain relevant information that is not visible in 2D data. Instead of 3D scans, estimated depth maps based on a 2D input image can be used with the advantage of a simple setup and a short recording time. Determining a 3D reconstruction from a single input image is a difficult task for which many algorithms and methods have been proposed in the past decades. In this work, three deep learning methods, namely stacked autoencoder (SAE), generative adversarial networks (GANs) and U-Nets are investigated, evaluated and compared for 3D reconstruction from a 2D grayscale image of laser-welded components. In this work, different variants of GANs are tested, with the conclusion that Wasserstein GANs (WGANs) are the most robust approach among them. To the best of our knowledge, the present paper considers for the first time the U-Net, which achieves outstanding results in semantic segmentation, in the context of 3D reconstruction tasks. Unlike the U-Net, which uses standard convolutions, the stacked dilated U-Net (SDU-Net) applies stacked dilated convolutions. Of all the 3D reconstruction approaches considered in this work, the SDU-Net shows the best performance, not only in terms of evaluation metrics but also in terms of computation time. Due to the comparably small number of trainable parameters and the suitability of the architecture for strong data augmentation, a robust model can be generated with only a few training data.

## 1. Introduction

The industry 4.0 megatrend is driving the digitization of production. It describes the intelligent networking of machines and processes for the industry with the help of information and communication technology [1]. A driving force of digitization is machine learning (ML), which is considered a key technology in data-driven industries.

In the field of industrial quality inspection, several applications based on ML are known. Unlike big data applications, the models are created based on data sets with a limited number of samples, especially in the field of research [2,3]. The reason is that the acquisition of training data often involves a series of experiments and therefore is associated with high efforts and costs. Ref. [4] used deep learning to perform quality inspection of hairpins. Hairpin technology is a winding design for stators in electric motors. In contrast to coil winding, an increased slot filling ratio is achieved [5]. At low to medium speeds, hairpin technology shows improved efficiency, allowing a reduction in motor size. In addition, this design alternative shows improved thermal behavior [6,7]. For quality inspection, ref. [4] analyzed and compared different architectures of convolutional neural networks (CNN). Furthermore, 3D scans or 2D grayscale images were used as input to the CNN and the results were evaluated. Thereby, the classification accuracy obtained with 3D scans was greater than that obtained with 2D grayscale images. This can be explained by the fact that the height values contained relevant information that could be used to detect certain error cases. By obtaining the depth map from a 2D image, an increased hardware complexity, as well as calibration effort or often longer acquisition times in the process could be saved. Despite the advantages mentioned and the potential of 3D scanning, there are still no approaches to generate 3D reconstructions from 2D grayscale images of industrial components, such as hairpins.

In other fields, except industrial applications, 3D reconstruction is an important area of research, e.g., in the estimation of surface profiles of human faces [8,9]. Various research approaches address the problem of constructing the most accurate 3D representation possible from a single image representing an object in two dimensions. The reconstruction of a 3D shape from a 2D image is an ill-posed problem since there is no unique solution. ML-based methods reconstruct 3D objects by learning the relationship between 2D images and 3D geometry. This approach has attracted a lot of interest because it avoids time-consuming reconstruction methods. Various approaches and network architectures can be used to solve the problem. One approach is a stacked autoencoder (SAE) [8]. Another promising approach to reconstruct the height information is the use of generative adversarial networks (GAN) [9,10,11]. In this work, the suitability of SAE and GANs for 3D reconstruction of industrially recorded hairpin data is investigated.

Based on the excellent results of the U-Net [12] in the field of semantic segmentation in medical technology and industrial usage [13], to the best of our knowledge this work investigates for the first time the potential of this method for 3D reconstruction problems. A modification of the U-Net, namely the stacked dilated U-Net (SDU-Net) [14], uses dilated convolutions with the advantage of a larger receptive field. The use of reconstruction algorithms in the industry requires favorable use of computational resources for efficiency reasons, which complicates the application of computationally intensive network architectures. The U-Net and the SDU-Net represent promising solutions in terms of resource utilization due to their small size and complexity [15,16].

## 2. Related Work

Three-dimensional reconstruction methods are divided into classical and learning-based methods. While classical methods deal with shape and image characteristics such as reflection, albedo or light distributions, deep-learning-based methods use complex network architectures to learn the correlations between 2D and 3D data.

Classical 3D reconstruction approaches are shape from shading (SFS) [17], structure from motion (SFM) [18], multiview stereo (MVS) [19] or shape from polarization (SFP) [20,21,22]. SFS reconstructs a shape based on the variation of shading, assuming a single point light source and Lambertian surface reflectance, where the brightness of an image pixel depends on the light source direction and the surface normal. Nevertheless, these assumptions are not always true for real images. SFM and MVS reconstruct a 3D object from several images taken from different known viewpoints. In addition to the sufficient number of images, these approaches moreover need the correspondence of features in the images to calculate the 3D shape. SFP is based on the principle that the degree of polarization of the light reflected from an object depends on its reflection angle. Hence, by measuring the degree of polarization, the surface normals of an object are determined. Originally, SFP was developed for transparent or reflective dielectric objects [23,24]. Subsequently, it was extended to highly reflective metallic objects [25]. However, unfavorable to this method is the necessity of a polarization camera in the production process.

Deep-learning-based methods have shown encouraging performances in a variety of computer vision problems, including 3D reconstruction [26,27,28,29,30]. Yet, many approaches are difficult to integrate into existing industrial processes as new camera or lighting setups are required. A deep-learning-based method that overcomes the mentioned drawbacks is the autoencoder. Zhang et al. [8] proposed to reconstruct 3D surfaces of human faces from corresponding 2D images with a stacked autoencoder (SAE). Thereby, low-dimensional features of the 2D and 3D images were learned separately and connected with another network. The result was a deep neural network that had a 2D image as input and a 3D shape as output.

A special deep learning structure called generative adversarial network (GAN) [31] has received a lot of attention because it can generate realistic images, whose property is also interesting for the task of 3D reconstruction. The network structure consists of two separate networks: a generator and a discriminator. The generator is trained to create realistic images while the discriminator tries to distinguish these images from real ones. A variant of GANs is a conditional generative adversarial network (CGAN) [32]. Many problems in the field of image processing and computer vision such as segmentation [33], super resolution [34], corner-to-object [11] or single-view 3D reconstruction [9] can be described as an image-to-image translation task [11] and solved with CGANs. Training GANs is difficult since it is unstable and highly dependent on the choice of parameters [35]. To improve convergence, alternatives have been suggested [36,37,38,39]. Arjovsky et al. [37] proposed the Wasserstein distance for their GAN structure, named Wasserstein GAN (WGAN), with improved stability. This architecture was used, among other things, to obtain a 3D depth map from a 2D image of human faces [10,40].

To the best of our knowledge, our method uses for the first time the U-Net network architecture proposed by Ronneberger et al. [12], which is based on convolutional neural networks (CNN) [41], for the use case of 3D reconstruction. The network can perform efficiently on augmented data, which is especially important in industrial research, as usually few data are available. The U-Net architecture consists of a contracting path to capture the context and a symmetric extension path connected by skip connections which realizes precise localization. Many variants such as attention U-Net (AttU-Net) [42], recurrent residual U-Net (R2U-Net) [43] or nested U-Net (U-Net++) [44] have been proposed. These variants deliver better results than the classic U-Net for special applications, but typically with greater computational effort. Since the convolutions of the U-Net have a limited receptive field, ref. [45] introduced dilated convolutions in the U-Net, where the dilation rate was increased while the resolution was decreased. On the other hand, ref. [46] pointed out that this approach was unfavorable for small objects. To exploit the superior segmentation performance of the U-Net and at the same time overcome disadvantages such as small receptive fields, Wang et al. [14] proposed a more efficient U-Net variant, named stacked dilated U-Net (SDU-Net). Thereby, the input layer was processed at different resolutions using multiple dilated convolutions and all results were combined as input for the next layer.

## 3. Materials

As for industrial research usually no publicly accessible data sets are available, the required data for this work were recorded in a laboratory. Hairpins are promising in the field of winding constructions of electric motors. They consist of two straight copper flat wire elements that are inserted into stator slots and then welded together in pairs with a laser. Already-welded pairs of copper wires were used for data acquisition. The images of 953 hairpins were taken from above, as this perspective allows the integration into the existing industrial process. The principle of optical coherence tomography (OCT) was used to record the 3D data. Based on the height information, features of the components can be detected that are not visible in the 2D camera image. The disadvantage of OCT is the recording time, which is significantly longer than that of a conventional 2D camera for industrial applications.

### 3.1. Experimental Setup

**Figure 1:**
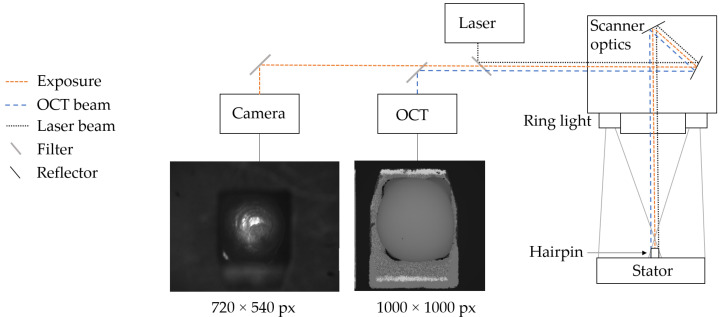
Experimental setup for hairpin data acquisition.

Figure 1 shows the experimental setup for the data acquisition of hairpins. Since the setup had to be integrable into industrial processes, the already-existing hardware arrangement was used. The hairpins were inserted into the stator. A laser welding process connected the two copper wires. The weld quality is influenced by parameters such as material quality, beam deviations, focus position and environmental factors. These process deviations lead to potential welding defects [47]. Using two mirrors, a programmable scanner optic could position the laser beam at any given position within a processing field. In addition, the images were illuminated with a ring light. The advantage was the direct mounting of the LED ring light on the scanner optics, which allowed an easy integration into the production process.

The 2D intensity images of the hairpins were recorded with a VCXG-15M.I industrial camera from Baumer (Frauenfeld, Switzerland), which is based on CMOS technology. The images had a resolution of 720×540 pixels, with a pixel pitch in the x- and y-directions corresponding to 18 μm each. To obtain the height maps of the hairpins, many line scans were performed according to the principle of OCT [48]. These were then combined to obtain an overall height map of the component. The lateral resolution of the height map was 1000×1000 pixels, with a step size of 7.5 μm. The displayed height information was recorded in increments of 11.7 μm, with the sensor covering a measurement range of approximately 12 mm.

### 3.2. Preprocessing

In addition to the experimental setup, Figure 1 shows a raw image of the industrial camera and the OCT. The height values from the OCT scan were converted to a grayscale image for further processing. Each pixel of the image represented a scan point from the height map. The height values were scaled to 256 gray values, resulting in increments of 46.8 μm. This loss of accuracy in the elevation data did not affect a downstream quality assessment based on the elevation data. The height difference of the hairpins was in the millimeter range and the error cases showed more significant height deviations than 46.8 μm. Such small deviations were not relevant, so that scaling to 256 values could be performed. Among others, it was shown in [4] that a meaningful quality assessment can be made with this simplification.

Because OCT is prone to artifacts and noise, unwanted disturbances occur in the 3D images. For this reason, preprocessing steps of the 3D data were applied. The opening in the stator surrounding the hairpin was outside the measuring range of the OCT. For this reason, noise occurred there in the images, which was filtered out in a preprocessing step. This was done by using semantic segmentation to detect the pin area in the image. Through the mask predicted by the model, the image was filtered to the relevant area. In addition, artifacts on the hairpin surface were eliminated by outlier detection. The artifacts were caused, on the one hand, by contamination of the optics, but could also result from measurement errors of the OCT. All artifacts had in common that they were outliers of a few pixel values that deviated from their local environment. It could be physically excluded that such artifacts were caused by the welding process, for example by spattering. To detect the outliers, a distance-based algorithm was used. Thereby, the pixel values were compared with the respective values of the neighboring pixels and replaced by the average of the neighborhood if the deviation was too large. Due to the fact that the mentioned preprocessing was only applied to 3D data, it did not affect the industrial process for 3D reconstruction, and therefore only the 2D images were used. The preprocessing depended on the sensor technology used for data acquisition and aimed to obtain the best possible ground truth data of the 3D images in training.

After preprocessing the 3D data, a mapping algorithm was used because a 3D reconstruction from a 2D image requires image pairs that are aligned identically in terms of translation, rotation and scaling. As described in the experimental setup, the acquired data had different sizes and scales. The performed mapping used the image area corresponding to the OCT scan. Even if a larger area was visible in the camera image, the height information was only available for the area of the OCT scan. Thereby, the corresponding area in the camera images was defined manually and the images were cropped to this size. The resolution, on the other hand, was determined by the 2D images. Thus, the resolution of the OCT scans was scaled down accordingly. This also entailed a loss of accuracy, but this was tolerable for our application. The component had a smooth surface structure that did not show drastic changes within 7.5 μm. Therefore, scanning at a distance of 18 μm was sufficient.

**Figure 2:**
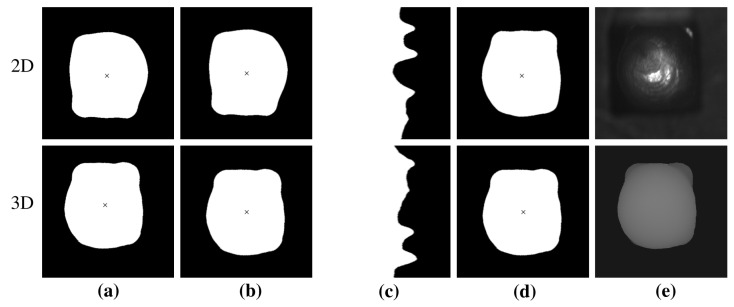
Mapping of the 2D and 3D images of a hairpin. The images of the upper row belong to the 2D image, the lower row to the 3D height profile. The raw data of the considered pin can be seen in Figure 1. (**a**) Masks of the 2D and 3D images learned with a neural network and their centers. In (**b**), the centers of both masks are translated at the center of the image. The images are transformed into polar coordinates, which can be seen in (**c**). In (**d**), the 2D mask is rotated. (**e**) Result of the mapping.

To determine the transformation, corresponding locations of the images were detected. For the existing data, the shape of the pins was suitable because it was recognizable in the intensity images as well as in the height profiles. For this, a semantic segmentation to locate the regions of the pins was used, with binary masks trained on the 3D and 2D images. Then, the transformation to turn the mask of the 2D image to the mask of the 3D image was determined and applied to the intensity images. The procedure is illustrated in Figure 2. First, the centroid of the pixels belonging to the pin was determined for both masks. This was assumed to be the point around which the 2D mask was rotated to be translated to the 3D mask. To determine the rotation, the masks were transformed into polar coordinates. The representation in polar coordinates allowed the application of correlation. From the maximum correlation, the translation of the polar coordinate images was derived. From the translation $t_{\mathrm{pol}}$ of the polar coordinates, the rotation angle *r* was derived according to

(1) $$r=\frac{t_{\mathrm{pol}}}{l}\;\cdot \;360^{\circ }$$

where *l* is the length of the image. In a postprocessing step, a correction of the translation was made using the correlation to the 3D mask. It has to be noted that in an automated process, the mapping step is not necessary, since there exists a uniform calibration.

### 3.3. Data Augmentation

Data augmentation (DA) is essential to teach invariance and robustness properties to a neural network, especially when few training data are available [49]. Like most datasets in the field of industrial research, the recorded dataset, consisting of 953 samples, was small for applications of machine learning. For this reason, strong DA was essential for the success of 3D reconstruction algorithms. In series production, hairpins are not always centered and rotated in the same way. Furthermore, some pins have a modified geometry. This motivated the application of rotation, translation, mirroring, scaling and shearing to the training data. This made the trained model more robust and avoided the need to retrain it in case of small changes. As the training of GANs is problematic, the successfully used DA from [11] was applied for the GANs. Depending on the training parameters, a different number of generated data samples was used for each 3D reconstruction method. Table 1 shows the used dataset, where 80% of the data were used for training and 20% for testing.

## 4. Methods for 3D Reconstruction

To investigate the potential of 3D reconstruction algorithms for industrial data, three different approaches were used. All methods originated from the field of machine learning but used different structures and training procedures.

### 4.1. Stacked Autoencoder

An autoencoder is a neural network that preserves the information between input and output intending to produce meaningful features contained in low dimensionality. The network consists of two parts: an encoder function l=f(x) and a decoder $\hat{x}=g\left(l\right)$. A stacked autoencoder (SAE) differs from a traditional one in the way of training. It is characterized by the fact that each layer is trained separately. Afterward, they are stitched together. To reconstruct a 3D representation of a 2D image, low-dimensional feature spaces for the 2D images and the 3D data are learned separately using a stacked autoencoder. Then, the two feature spaces are connected with a fully connected layer. This results in a network whose inputs are 2D images and whose outputs are the corresponding 3D representations. After merging, the parameters are probably not optimal. The network can be fine-tuned with a gradient descent method and backpropagation, as explained in [8].

### 4.2. Generative Adversarial Networks

Generative adversarial networks (GANs) consist of a generator and a discriminator. The generator performs a mapping from a random noise vector z to an output image y,G:z→y and the discriminator tries to distinguish them from real training data. In contrast, conditional generative adversarial networks (CGANs) learn a mapping of an observed 2D image x and a random noise vector z to a 3D output y, G:{x,z}→y. The objective of a CGAN is expressed as

(2) $$L(G,D)=E_{y∼p_{data}\left(y\right)}\left[\log D\left(y|x\right)\right]+E_{z∼p_{z}\left(z\right)}\left[\log\left(1-D\left(G\left(z|x\right)\right)\right)\right],$$

where *G* tries to minimize this objective against an adversarial *D* that tries to maximize it [11]. The L1 or L2 distance measure can be added to the objective function to prevent the generated images from being too far away from the actual values [11]. The final problem is

(3) $$G^{*}=\arg\min_{G}\max_{D}L\left(G,D\right)+\lambda L_{L1|L2}\left(G\right).$$

Training GANs is problematic due to vanishing or exploding gradients, overfitting or a nonconverging model. Wasserstein GANs (WGAN) overcome some of the problems of regular GANs. Arjovsky et al. [37] proposed the Wasserstein distance, which is the distance between two probability distributions *p* and *q* of a region as

(4) W(p,q)=inf(E[d(X,Y)]),

where E is the expected value of all joint distributions of the random variables *X* and *Y*, and d(·) is the absolute-value function. The infimum in (4) makes it difficult to obtain a solution. To approximate it, a K-Lipschitz condition and weight clipping [37] were introduced.

### 4.3. U-Nets

The U-Net [12] consists of a contraction path to capture the context and a symmetric extension path for precise localization. The two paths are connected by skip connections, with the advantage that information from higher layers can be used directly. Consequently, not all information has to pass the deepest layer of the network. A disadvantage of the U-Net is that the used convolutions have a small receptive field. Hence, the stacked dilated U-Net (SDU-Net) [14] adopts the general network architecture of the U-Net, modifying the encoder and decoder operation. The two standard convolutions of the U-Net are replaced by one standard convolution followed by four dilated convolutions that are concatenated. Compared to the U-Net, the SDU-Net maps different scales and larger receptive fields with fewer parameters. Since for the 3D reconstruction of hairpins, local areas such as punctual elevations or splashes as well as larger areas such as the shape of the pin are important, the SDU-Net is promising. This work investigated for the first time the potential of the U-Net for 3D reconstruction problems, motivated by excellent results of this method in the field of semantic segmentation. The difference between the 3D reconstruction task and semantic segmentation is as follows: instead of assigning a class label to each pixel, the corresponding height value is used as a label. This task is more challenging compared to semantic segmentation insofar as the number of possible values is extended to the number of gray values and thus includes up to 256 values depending on the height profile.

## 5. Network Structures and Training Details

A total of six 3D reconstruction configurations were examined. Table 2 shows the number of parameters of the different methods. Thereby, the SAE had the most, which was caused by the exclusive use of fully connected layers. The architectures of the classical U-Net and the SDU-Net had fewer parameters than the other methods, with the SDU-Net having the fewest. The 3D reconstruction methods were implemented in Python using Keras and Tensorflow. The training processes were run on an Nvidia GeForce RTX 2080Ti GPU card. In the following, the detailed network architectures and training details of the different methods are described.

### 5.1. Stacked Autoencoder

The neural network was constructed by linking the encoder and decoder subspaces as well as the mapping function between the two subspaces. The input and output resolutions were 256 × 256 pixels. Larger resolutions are problematic due to GPU memory constraints. The images were scaled to a value range of [0, 1]. The exact layer structure of the SAE was 256 × 256-1000-100 for the encoder and 100-2000-256 × 256 for the decoder. A layer consisting of 5000 neurons fully connected the 2D and 3D subspaces. The layers were trained separately. The activation function at the output of the last layer was a sigmoid function. The sigmoid function scaled the output data in the range [0, 1]. Since this also corresponded to the range of our input data, this helped to make the learning process more stable.

In training, the mean squared error (MSE) was used as the loss function. An Adam optimizer with $\beta_{1}=0.9$, $\beta_{2}=0.999$ and a learning rate of $l_{r}=1\times 10^{-4}$ was applied. The first layer of the encoder and those of the decoder were trained for 4000 epochs. The deeper layers were trained for 800 epochs since the number of parameters of the network to be optimized was smaller. It was trained with 800 steps per epoch and a batch size of 1.

### 5.2. Generative Adversarial Networks

With the choice of a generator network, a discriminator network and a loss function, the construction of a variety of different GAN structures is possible. Three of them were analyzed in this work and are listed in Table 3. The input dimension of the generator and discriminator as well as the output dimension of the generator was 256 × 256 pixels. As in [11], the images were scaled to a value range of [−1, 1]. The generator of all three configurations used the modified U-Net structure according to [11]. Configuration I used the PatchGAN as in [11] as a discriminator. Configurations II and III used a conditional version of the Wasserstein GAN loss function with a deep convolutional GAN (DCGAN) [50] as a critic. CD*k* denotes a convolutional–batch normalization–dropout–ReLU layer with *k* filters and a dropout rate of 0.3. The architecture of the DCGAN was CD64–CD128 without batch normalization. The convolutions were of dimension 5 × 5 with a step size of 2. The output of the last layer was flattened. A final fully connected layer resulted in the one-dimensional output of the critic. A leaky ReLU‘ with α=0.3 was used as the activation function.

To train the networks, the standard approach from [31] was applied. An Adam optimizer with a learning rate $l_{r}=2\times 10^{-4}$ and momentum parameters $\beta_{1}=0.5$ and $\beta_{2}=0.999$ was used. The networks were trained from scratch. The weights were taken from a normal distribution with a mean of 0 and a standard deviation of 0.02. The networks were trained twice for 500,000 iterations with a batch size of 1. In the objective function, λ=100 was chosen as well as the L1 or L2 distance measure, depending on the configuration. The weight clipping factor of the WGAN was 0.01.

### 5.3. U-Nets

**Figure 3:**
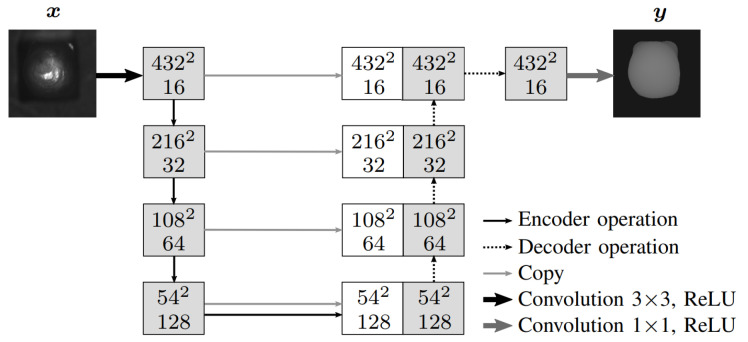
U-Net structure for 3D reconstruction. Input is the 2D gray-scale image of a hairpin *x*; output is the 3D reconstruction *y*. Boxes represent feature maps, where the first number indicates the spatial dimension and the second the number of channels. White boxes represent copied feature maps, which are linked to the decoder maps in the expanding path.

In this work, one architecture of the U-Net and one of the SDU-Net were investigated. Table 4 shows the two configurations to be examined. The dimension of the grayscale images at the input as well as the reconstructions at the output was 432 × 432 pixels. The images were scaled to a range of values of [0, 1]. Configuration I used a classic U-Net architecture. CPD*k* denotes a convolutional–batch normalization–max pooling–dropout–ReLU layer with *k* filters. CUD*k* contained a convolution–batch normalization–upsampling–max pooling–dropout–ReLU layer. Batch normalization is not present in the classic U-Net. The former was added to obtain a faster and more stable network. The encoder architecture was C16–CPD16–C32–CPD32–C64–CPD64–C128–CPD128–C256. The decoder structure was CUD256–C128–CUD128–C64–CUD64–C32–CUD32–C16–C16. The layers were connected with skip connections. All convolutions had the dimension 3 × 3 with a step size of 1. For upsampling, we used a convolution operation that used trainable weights to determine the optimal procedure for the upsampling step. The upsampling factor was 2. The activation function was a ReLU. The output layer used a sigmoid function to scale the output to [0, 1]. Configuration II employed the SDU-Net. It was examined whether a sufficiently good reconstruction could be obtained based on only a few training examples, which is advantageous for industrial applications. PsdC*k* denotes the encoder operation of the SDU-Net, consisting of a max pooling–sdConvolution–ReLu layer with the filter numbers *k*. The architecture of the encoder was PsdC16–PsdC32–PsdC64–PsdC128. In the first layer, the max pooling operation was omitted. UsdC*k* contained the decoder operation of the SDU-Net, consisting of a upsampling–sdConvolution–ReLU layer. The architecture of the decoder was Usd128–UsdC64–UsdC32–UsdC16–C1. Three skip connections between encoder and decoder were used. All convolutions had the dimension 3 × 3. An sdC-block consisted of one standard convolution and four dilated convolutions, with the filter numbers k/2, k/4, k/8, k/16 and k/16, respectively. For the stacked dilated convolutions, dilation rates of 3, 6, 9 and 12 were used. The step size was 1. The last convolution layer reduced the number of channels to 1. Here, the activation was a sigmoid function. The architecture of the U-Net configuration is illustrated in Figure 3. The feature maps shown as boxes consisted of the sdC-block instead of the two consecutive convolutional layers in the SDU-Net.

For the training of configurations I and II, the MSE was used as a loss function. An Adam optimizer with $\beta_{1}=0.9$, $\beta_{2}=0.999$ and a learning rate of $l_{r}=1\times 10^{-3}$ was applied. The learning rate was reduced as training progressed. Both U-Net configurations were trained with a batch size of 6 for 600 epochs with 500 steps per epoch.

## 6. Results

To compare the effectiveness of the trained neural networks in estimating 3D reconstructions, two accuracy metrics were used. These metrics compared the estimated 3D reconstruction with the true height map of the corresponding 2D input image. To obtain a meaningful comparison of the different methods, pixel-based metrics were used. This evaluation was adapted from the pixel-based loss function used in training. In training, the pixel-wise loss function had the advantage that each pixel could effectively be considered as an individual training sample, thus increasing the effective number of training images and preventing overfitting [51].

The pixel-wise mean absolute error (MAE) was calculated for each test image as

(5) $$E_{\mathrm{MAE}}=\frac{1}{l\;\cdot \;w}\sum_{x,y\in \Omega }e\left(x,y\right)$$

where *l* and *w* correspond to the length and width of the image. The expression e(x,y) is the absolute error of one pixel resulting from

(6) $$e_{\mathrm{abs}}\left(x,y\right)=|h_{r}\left(x,y\right)-h_{l}\left(x,y\right)|$$

or

(7) $$e_{\%}\left(x,y\right)=|\frac{h_{r}\left(x,y\right)-h_{l}\left(x,y\right)}{h_{\max}}|\;\cdot \;100.$$

where $h_{r}\left(x,y\right)$ is the height value calculated by the reconstruction algorithm and $h_{l}\left(x,y\right)$ is the corresponding true height value. Equation (6) gives the MAE as an absolute value. Equation (7) specifies the MAE as a percentage by referring to the maximum occurring height value $h_{\max}=7000\mu m$. To calculate the average MAE of all test samples, the mean value of the individual MAEs was determined. The root mean squared error (RMSE) between the reconstruction and the ground truth elevation profile was calculated as

(8) $$E_{\mathrm{RMSE}}=\sqrt{\frac{1}{l\;\cdot \;w}\sum_{x,y\in \Omega }{\left(h_{r}\left(x,y\right)-h_{l}\left(x,y\right)\right)}^{2}}.$$

### 6.1. Different Network Architectures

**Figure 4:**
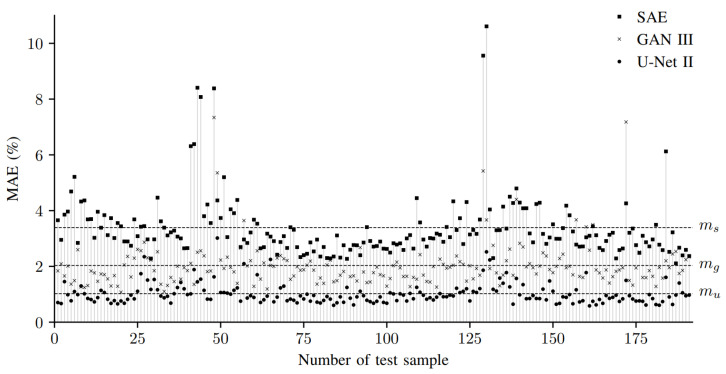
MAE of the 191 test samples. The MAE of three methods over the test samples is shown. $m_{s}=3.390\%$ is the mean MAE of the SAE, $m_{g}=2.033\%$ that of the WGAN with L2 norm and $m_{u}\;=\;1.021\%$ that of the SDU-Net (Table 5).

**Figure 5:**
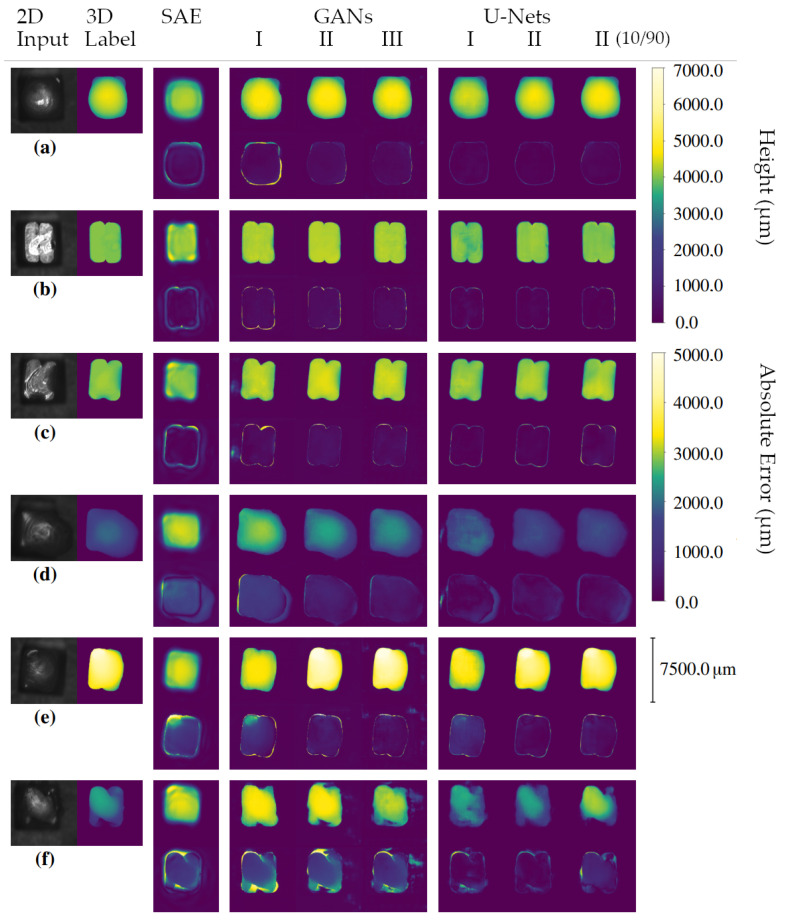
Three-dimensional reconstruction results of test input images. The reconstruction results of different input hairpins are shown: (**a**) good welding, (**b**) hairpin not in focus of laser, (**c**) welding with too little power, (**d**) welding with too much power, (**e**) hairpin with offset of copper rods and (**f**) copper rods without removed insulation. In each case, the first line shows the reconstruction and the second line shows the absolute error between label and reconstruction. The test sample numbers from (**a**–**f**) are 1, 47, 53, 130, 44 and 48. Figure 4 thus allows the determination of the MAE.

Table 5 shows the test results of the examined 3D reconstruction algorithms. It can be seen that the GANs outperform the SAE. Accordingly, the WGANs show better results than the CGAN. The U-Net-based 3D reconstruction approaches proposed in this paper outperform the other two methods by a large margin. The SDU-Net shows the best performance. Figure 4 presents the MAE of the individual test samples of the SDU-Net, the best performing GAN, namely, the WGAN with L2 norm, and the SAE. The MAE of the respective method is shown as a horizontal line. On closer inspection, it can be seen that there are very few test samples where the WGAN performs better than the SDU-Net. The proportion is only 2.618%. In Figure 5, the 3D reconstruction results from six hairpins of the test data set are given. The 2D input images, the 3D ground truth images as well as the results of the six neural networks are shown. The reconstruction of the SAE suggests the shape of the hairpin. Deviations on the pin surface are visible. There are no sharp edges but smooth transitions into the background. Characteristic features such as the offset of the pin from (e) are not learned. The CGAN shows high differences at the edges of the pins. It produces realistic-looking reconstructions. However, they partly deviate from the ground truth label. Looking at the difference images, it can be seen that the U-Net-based algorithms achieve the best results. Among them, the U-Net II stands out, for example, because the offset of the pin from (e) is reconstructed.

### 6.2. Number of Training Data

Since the industry usually requires working with a very small dataset, further experiments were conducted with a reduced size of the training data. Based on the best performance and the smallest number of model parameters, the SDU-Net configuration was used for the experiments. The model architecture lends itself to the use of strong data augmentation, which is necessary for robust prediction of new datasets with further reduction in training data. Furthermore, compared to SAE and GAN, the model does not oversimplify by drawing soft transitions or creating reconstructions with little relation to the input image. Its performance was checked when the number of training samples was reduced to 60%, 40%, 20% and 10% of the available data. The network and training parameters were defined in the same way as for the U-Net II configuration. The results were obtained by 20 different random train–test splits. The averaged result is shown in Table 6.

The results show that the performance of the SDU-Net decreases when the number of training data is reduced. Reducing the size of the training dataset from 80% to 10% of the available data degrades the average MAE on the test set by 14.7 μm. However, all results are still better than those of the GANs and SAE, whose results are shown in Table 5. With the best GAN method, we achieved an average MAE of 142.2 μm on 762 training samples and 237.3 μm with the SAE.

**Figure 6:**
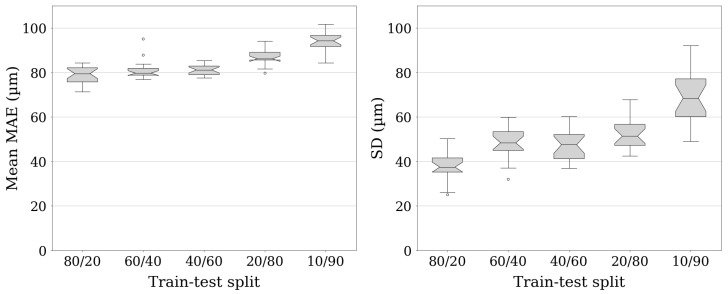
Distribution of the mean MAE and SD of the 3D reconstruction algorithm for different train–test splits. Validation of the U-Net II performance under different proportions of data in the training and testing part for 20 random splits each.

Furthermore, the variance of the average MAE within each train–test proportion shown in Figure 6 suggests that the composition of the training set has an impact on model performance. Pins from different defect classes have different geometries and height profiles, as seen in Figure 5. The differences between hairpins can be so significant that they must be considered during training. If only the features of very similar parts are learned, the reconstruction of divergent geometries may become inaccurate. Therefore, random splits lead to a worse average result than a representative training dataset, as also shown by the comparison with Table 5. An unbalanced training dataset is also a possible explanation for poor performance with less training data. Using less data makes it more difficult to capture all of the variance. This increases the average MAE and standard deviation in tests, since unknown geometries cannot be calculated accurately.

## 7. Discussion

In our experiments, the superiority of GANs and U-Nets over the SAE became clear. One reason why the SAE performed below the expectations from [8] might be the images used. The research in [8] used synthetic face images. For real industrial data with textures and shading, the SAE led to poor results. Furthermore, autoencoders work as lossy compressors; in each layer, a considerable amount of information is eliminated. This was observed by comparing the output with the input image after passing through one compression layer. Moreover, in the present context, the exclusive use of a sigmoid activation in [8] was a hindrance. Excluding the output layer, it was better not to restrict the values of the deeper layers to [0, 1]. In contrast to the architecture of the SAE, the U-Net with its skip connections delivered a robust structure for the 3D reconstruction task of hairpins. Thus, it enabled the generator network of GANs as well as the U-Net-based approaches to take into account information on different processing levels. With the help of convolutional layers in the contraction path, the focus was continuously shifted from the localization to the content of an image. Consequently, for example in the first skip connection, the information of the pin’s location could be passed directly to the output. The SDU-Net was more efficient than the usual U-Net due to the use of stacked dilated convolutions. It showed higher robustness against local fluctuations and reconstructed the entire hairpin better. This is consistent with the fact that the SDU-Net has a wider receptive field.

Both the generator of the GANs and the U-Net-based approaches used a U-Net architecture as their foundation. One main difference lay in the way of training. The U-Net-based approaches optimized the parameters by training the network end-to-end whereas GANs trained two adversarial networks. An advantage of the second type of training was that the generated images looked realistic, for example, the sharp edges of the hairpins were learned. However, hard edges implied that the algorithm had to decide on the positions for edge pixels, even if it was not sure. In contrast, with the SDU-Net, it could be seen that softer edges were tolerated and generated during training. However, these softer edges still led to large deviations of the reconstruction algorithms at the edges of the hairpins. One possible reason for this was that the data pairs were not mapped exactly. That possibly resulting inconsistency in the mapping of the image pairs ultimately may have led to inaccuracies and uncertainties in the neural network concerning the localization of the edges. A serious problem of the SAE was the significant number of parameters caused by the fully connected layers. Without any convolutional layers, it was noticeable that training with images in larger resolutions was a gigantic workload. For example, the first layer of the implemented SAE had over 65 million parameters. These were over 400 times more than what the entire SDU-Net had. A smaller number of parameters goes hand in hand with a shorter training time as well as a smaller memory requirement of the neural network. Furthermore, the calculation time of the 3D reconstruction is a decisive factor for the integration into the industrial manufacturing process. The use of stacked dilated convolutions reduced the number of parameters of the SDU-Net by 93% compared to the U-Net. To be able to use the 3D reconstruction algorithm for quality monitoring directly on the production line, the prediction time must not significantly affect the cycle time. In addition, for execution on existing hardware, a cost-effective use of computational resources is required for efficiency reasons, which precludes the use of computationally intensive network architectures. Therefore, the U-Net-based 3D reconstruction algorithms proposed in this work are the most suitable solution.

## 8. Conclusions

In this work, three different deep-learning-based 3D reconstruction methods, namely SAE, GANs and U-Net-based approaches were examined. The SAE learned nonlinear subspaces from both 2D images and the corresponding 3D scans and linked them. The results showed that the lossy compression property of the SAE was evident in the considered hairpin data. Furthermore, three different structures of GANs were trained. Thereby, the Wasserstein GAN (WGAN) was superior to the CGAN in terms of stability and robustness and solved the problem of convergence during training.

To the best of our knowledge, the present paper was the first to use a U-Net approach in the context of 3D reconstruction tasks. This work concludes that U-Net-based algorithms outperform the SAEs and GANs. Two different U-Net architectures were applied to reconstruct the height profile of hairpins from a single 2D grayscale image, all of which performed better than the SAE and GANs. Among the architectures, the stacked dilated U-Net (SDU-Net), which was based on dilated convolutions, proved to be the structure with the lowest error rates concerning two different evaluation metrics. The use of this convolutional structure provided not only the best 3D reconstruction but also the lowest parameter count of 162,423 among the committed methods. In summary, the SDU-Net not only showed the best performance in terms of the two evaluation metrics but also in terms of model size and computation time. Thus, the SDU-Net is the most suitable for industrial processes.

Furthermore, it could be shown for the investigated use case that the training data set could be strongly reduced. Among other things, this could be traced back to the small model architecture of the SDU-Net, which had only a few parameters. In addition, the data set in industrial processes is usually homogeneous and the learned features can be transferred well. Nevertheless, all contingencies should be covered in the training data set. Since some defect classes were only represented in a very small quantity of data, splitting 10% training data may have resulted in none of these images being included in the training process. This explained the slightly worse results than a split of 80/20.

Undoubtedly, the U-Net architecture is easily applicable to other 3D reconstruction tasks besides hairpin data. For this purpose, only a new model must be trained. In future work, the developed solutions will be integrated into the manufacturing process. Depending on the results, it may be necessary to modify the networks. Performing hyperparameter optimization may also be useful to improve results. Furthermore, the occurring variance in the data must be monitored. Due to the DA used in training, the model is already robust to slightly changed data. With longer production cycles, it could possibly happen that the data differ more from the training data due to the wear of tools or such. In this case, a post-training with data recorded over a longer time interval could be useful. Despite the superiority of the approaches based on the U-Net, GANs have the advantage of generating realistic-looking images. Combinations of the SDU-Net and GANs could bring out the strengths of both methods. It is conceivable, for example, to replace the generator structure of the GANs with an SDU-Net architecture. In this work, it was shown that the SDU-Net 3D reconstruction approach could be used to improve the quality inspection of hairpins. Consequently, industry 4.0 technologies can contribute to the optimization of electric motor production, with machine learning being the key technology.

## Figures and Tables

**Table 1:** Dataset for 3D reconstruction.

Database	$n_{\mathrm{train}}$	$n_{\mathrm{test}}$	$n_{\mathrm{all}}$	$n_{\mathrm{DA}\_\mathrm{SAE}}\;\left(10^{6}\right)$	$n_{\mathrm{DA}\_\mathrm{GAN}}\;\left(10^{6}\right)$	$n_{\mathrm{DA}\_U-\mathrm{Net}}\;\left(10^{6}\right)$
2D image	762	191	953	3.2	1.0	1.8
3D OCT scan	762	191	953	3.2	1.0	1.8

**Table 2:** Number of parameters of the implemented 3D reconstruction algorithms. The parameters of the GANs refer to the generator network.

Structure	Number of Parameters
SAE	197,981,736
GANs	54,419,713
U-Net	2,164,305
SDU-Net	162,423

**Table 3:** Configurations of GANs for 3D reconstruction.

Configuration	Generator	Discriminator	Loss Function
I	U-Net	PatchGAN	CGAN + L1
II	U-Net	DCGAN	WGAN + L1
III	U-Net	DCGAN	WGAN + L2

**Table 4:** Configurations of U-Nets for 3D reconstruction.

Configuration	Structure	Number of Filters
I	U-Net	16
II	SDU-Net	16

**Table 5:** Mean MAE and RMSE of the 3D reconstruction algorithms. In addition of the MAE, its standard deviation (SD) is calculated to indicate the dispersion in the test samples.

Structure	MAE (μm)	MAE (%)	SD (μm)	SD (%)	RMSE (μm)
SAE	237.3	3.390	82.5	1.179	471.0
GAN I	197.7	2.824	85.9	1.227	473.9
GAN II	174.5	2.492	63.1	0.902	339.2
GAN III	142.2	2.033	57.2	0.816	303.0
U-Net I	74.4	1.062	38.6	0.551	237.8
U-Net II	**71.4**	**1.021**	**26.1**	**0.373**	**229.4**

**Table 6:** Mean MAE of the 3D reconstruction algorithm SDU-Net with reduced number of training data. Different proportions of the data were used in the training part. In addition of the MAE, its standard deviation (SD) was calculated to indicate the dispersion in the test samples. The table shows the averaged values of 20 random train–test splits each.

$n_{\mathrm{train}}\;\left(\%\right)$	$n_{\mathrm{test}}\;\left(\%\right)$	$n_{\mathrm{train}}$	$n_{\mathrm{test}}$	MAE (μm)	MAE (%)	SD (μm)	SD (%)
80	20	762	191	78.8	1.126	37.2	0.532
60	40	572	381	81.1	1.158	48.1	0.687
40	60	381	572	81.0	1.157	47.1	0.673
20	80	191	762	86.9	1.242	52.2	0.745
10	90	95	858	93.5	1.336	68.7	0.981

## Data Availability

Not applicable.
